# Corticosteroid Injection for an Orthopedic Complaint in a Female with Gestational Diabetes

**DOI:** 10.1186/s40798-017-0115-y

**Published:** 2018-01-05

**Authors:** Palee Myrex, Lorie Harper, Sara Gould

**Affiliations:** Birmingham, USA

**Keywords:** Gestational diabetes mellitus, Blood glucose, Corticosteroid injection

## Abstract

A female with gestational diabetes presented with hip pain characteristic of meralgia paresthetica and trochanteric bursitis. She had similar episodes prior to pregnancy that were treated successfully with non-steroidal anti-inflammatory drugs (NSAIDs) and corticosteroid injections. However, NSAID use during pregnancy poses risks to the fetus and corticosteroids carry a risk of hyperglycemia, especially in those with diabetes. Unfortunately, all attempts made to treat her conservatively failed to improve her symptoms. The use of antenatal corticosteroids as an intervention for preterm labor has been documented, but to our knowledge, there are no published reports of corticosteroid injections for orthopedic complaints in someone with gestational diabetes. Review of her glucose log showed well-controlled levels, and subsequently, a corticosteroid injection was administered. Blood glucose levels were monitored for the next 48 h, and all measurements were within normal limits. The patient’s symptoms resolved, and she went on to vaginally deliver a healthy term infant without complications, suggesting that gestational diabetes should not be used as absolute criteria to withhold corticosteroid injections for orthopedic complaints.

## Key points

- Orthopedic complaints in pregnant patients can be difficult to treat as providers may be hesitant to employ certain traditional therapies in the gravid patient.
- Corticosteroid injections are often used to help fetal lung maturity during preterm labor, but corticosteroids have not been studied for their use to treat orthopedic complaints in the mother.
- Although corticosteroids carry the risk of hyperglycemia, gestational diabetes should not be used as absolute criteria to withhold corticosteroid injections for orthopedic complaints.

## Findings

### Case Report

A 32-year-old female recently diagnosed with gestational diabetes after an abnormal glucose challenge test (blood glucose level of 206 mg/dL at 1 h) presented to the sports medicine clinic at 30 weeks pregnant with lateral hip pain. She described the pain as a “burning that started in the lateral aspect of the left hip and radiated down the lateral aspect of the thigh.” The patient also reported lateral hip pain which was worse when rising from a seated position, climbing stairs, and lying on her left side. The pain was rated as a 2/10 in severity and was similar to the pain she had prior to pregnancy that was treated successfully with NSAIDs and a corticosteroid injection into the trochanteric bursa. Physical exam was notable for the absence of skin changes and absence of tenderness to palpation in the left groin but positive for tenderness to palpation in the lateral aspect of the thigh as well as directly over the greater trochanter. Range of motion testing was limited by her gravid habitus, but there was normal external and internal rotation of the left hip. Complete patient history and physical exam were indicative of meralgia paresthetica as well as trochanteric bursitis.

**Fig. 1:**
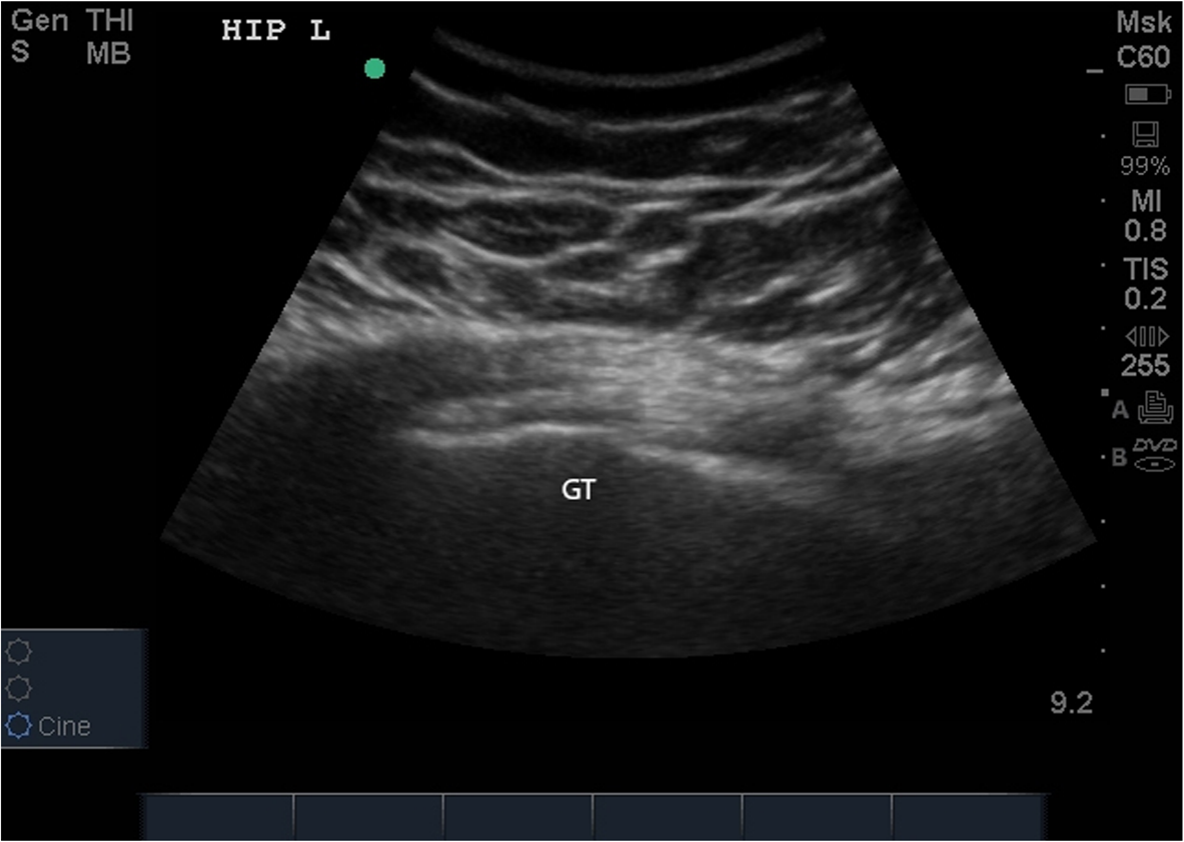
Ultrasound of the greater trochanter (GT) and inflamed bursa

Although her previous episodes had been treated successfully with NSAIDs and corticosteroid injections, NSAIDS are contraindicated in the third trimester of pregnancy and her gestational diabetes placed her at an increased risk of hyperglycemia from corticosteroid injections. Conservative measures such as physical therapy, education about weight loss, and instructions to wear loose clothing were recommended; however, the patient returned around 2 weeks later with no improvement in her symptoms. The patient’s daily blood glucose log was reviewed, and it showed an average fasting blood glucose level of 92 mg/dL and an average postprandial blood glucose level of 119 mg/dL. She was controlling her blood glucose by diet and exercise alone. Clearance was obtained by her OB/GYN to receive the corticosteroid injection, and subsequently, the patient received an ultrasound-guided injection of a sterile mixture containing 4 cc 0.5% marcaine and 40 mg methylprednisolone acetate into the left greater trochanteric bursa (Fig. 1). Blood glucose levels were closely monitored for the next 48 h, and all readings were within normal limits. At a 1-month follow-up, the patient reported that the pain was significantly improved, now rated as 0/10, and therefore, she had returned to her normal exercise routine. Approximately 6 weeks after the steroid injection, the patient vaginally delivered a 3000-g male at 38 weeks and 1-day gestation. There were no complications, and the baby tolerated delivery well, recording APGAR scores of 7 at 1 min and 9 at 5 min.

### Discussion

Our patient was experiencing meralgia paresthetica and trochanteric bursitis, both orthopedic conditions that can be related to the physiologic and anatomic changes of pregnancy [1, 2]. Glucocorticoids are potent immunosuppressive and anti-inflammatory drugs indicated for various orthopedic complaints including refractory meralgia paresthetica and trochanteric bursitis. However, a potentially serious complication of corticosteroid use, especially in a patient with gestational diabetes, is their propensity to cause hyperglycemia and possibly diabetic ketoacidosis [3, 4]. Diabetic ketoacidosis during pregnancy poses significant risk to the fetus as a single episode of diabetic ketoacidosis is associated with a 10–25% fetal loss rate [5]. Despite the risks associated with these drugs, they can still be used safely in pregnancy. A prime example is the use of antenatal corticosteroids to accelerate lung maturity in the fetus of a threatened preterm labor [6]. The benefits of improved fetal survival are so great that the National Institute of Health and Care Excellence (NICE) state in their clinical guidelines that “diabetes should not be considered a contraindication to antenatal steroids for fetal lung maturation [7].” NICE provides general guidelines for managing this subset of patients, yet they emphasize that each individual should be managed on a case-by-case basis.

Although the guidelines for administration of antenatal corticosteroids in threatened preterm labor are documented, there is little data about corticosteroid injections for orthopedic complaints in pregnant women, especially those with gestational diabetes [8]. As the prevalence of gestational diabetes is increasing, clinical scenarios such as the one with our patient are also likely to increase. Therefore, we felt it was necessary to share this report to draw attention to an important clinical complication and demonstrate the safety of an intervention that might otherwise be withheld during pregnancy due to concerns of the possible complications. Despite having gestational diabetes, our patient’s daily blood glucose levels were controlled and she tolerated the corticosteroid injection well. Her symptoms improved markedly, and there were no significant adverse effects on her blood glucose or subsequent impact on the baby. This absence of adverse effects may be due to non-fluorinated glucocorticoids, such as methylprednisolone, having minimal placental transfer [9]. In summary, all cases should be handled on an individual basis but gestational diabetes should not be an absolute contraindication for corticosteroid injections used to treat orthopedic injuries.

## Abbreviations

NICE: National Institute of Health and Care Excellence
NSAIDs: Non-steroidal anti-inflammatory drugs
